# Design and application of a web-based intelligent ophthalmic image analysis teaching platform

**DOI:** 10.3389/fmed.2026.1815698

**Published:** 2026-04-23

**Authors:** Junyi Chen, Yang Yang, Yuanzhuo Song, Zhiwei Huang, Xinwei Li, Biao Qu, Jiangzhong Wan, Lisha Zhong

**Affiliations:** 1 School of Medical Information and Engineering, Southwest Medical University, Luzhou, China; 2 School of Basic Medical Sciences, Harbin Medical University, Harbin, China; 3 School of Life Health Information Science and Engineering, Chongqing University of Posts and Telecommunications, Chongqing, China

**Keywords:** artificial intelligence, fundus photography, medical education, ophthalmic image analysis, optical coherence tomography, web-based platform

## Abstract

**Background:**

The rapid integration of artificial intelligence (AI) into ophthalmology has created new demands for interdisciplinary education that combines clinical image interpretation with intelligent analytical methods. However, structured teaching platforms that support end-to-end ophthalmic image analysis training remain limited. This study aimed to design and evaluate a web-based intelligent ophthalmic image analysis teaching platform and to assess its usability and educational effectiveness.

**Methods:**

A modular web-based platform was developed to support fundus photography and optical coherence tomography (OCT) analysis workflows, including image preprocessing, model training, performance evaluation, and AI-assisted interpretative guidance. A questionnaire survey (*n* = 121) was conducted to assess usability using the System Usability Scale (SUS) and competency ratings. In addition, a controlled teaching experiment involving 64 third-year undergraduate students compared learning outcomes between students receiving traditional instruction alone and those using the platform as a supplementary tool.

**Results:**

The platform successfully implemented a complete instructional workflow integrating clinical image cognition and AI-driven analysis. The median SUS score was 80.0 (70.0, 87.5), significantly exceeding the benchmark value of 68 (*p* < 0.001). Five self-assessed competency dimensions demonstrated mean scores ranging from 4.31 to 4.53 (maximum score = 5), all exceeding 4.0. In the controlled experiment, the experimental group achieved significantly higher scores in theoretical competence (78.62 ± 12.62 vs. 71.41 ± 13.03), practical competence (92.70 ± 4.18 vs. 86.45 ± 10.57), and comprehensive competence (87.34 ± 7.45 vs. 79.77 ± 10.94) compared with the control group (*p* < 0.05).

**Conclusion:**

The proposed web-based intelligent ophthalmic image analysis teaching platform demonstrated high usability and measurable educational benefits. Integrating clinically contextualized AI workflows into ophthalmic education may support competency development in intelligent ophthalmic education and training.

## 1 Introduction

Ophthalmic imaging has become an indispensable component of modern ophthalmology (1), playing a critical role in disease diagnosis (2), monitoring (3), and therapeutic decision-making (4). With the widespread clinical adoption of imaging modalities such as fundus photography and optical coherence tomography (OCT), large volumes of ophthalmic images are routinely generated, providing rich structural and pathological information for both clinical practice and biomedical research (5–7). These imaging technologies enable high-resolution visualization of retinal anatomy and disease-related alterations, thereby forming the foundation for image-based ophthalmic assessment.

In recent years, advances in artificial intelligence (AI), particularly deep learning, have substantially transformed ophthalmic image analysis (8–10). Intelligent algorithms have demonstrated strong performance in tasks including disease screening (3), lesion detection (11), severity grading (11), and anatomical segmentation (12), with increasing integration into clinical decision support systems (13). Consequently, ophthalmology has become an important application area of AI in medicine (14, 15). This rapid technological evolution has also generated new demands in medical education, emphasizing the cultivation of interdisciplinary talents who possess both ophthalmic domain knowledge and the capability to implement and evaluate intelligent algorithms on real-world medical image data (16). Ophthalmic images are characterized by complex imaging principles, fine-grained anatomical details, and strong reliance on domain-specific clinical interpretation (17). Meanwhile, AI-driven image analysis workflows, especially deep learning pipelines, are inherently abstract and multi-staged, involving data preprocessing, feature representation learning, model optimization, validation, and performance interpretation (18, 19). The limited interpretability of algorithmic processes further increases cognitive load and hampers conceptual integration across disciplines. Traditional lecture-based approaches, often centered on theoretical derivations and fragmented coding demonstrations, are insufficient to bridge the gap between algorithmic operations and clinically meaningful ophthalmic image interpretation (20). As a result, students frequently encounter difficulties in forming a coherent and interdisciplinary understanding of intelligent ophthalmic image analysis and in effectively transferring AI technology to authentic clinical imaging scenarios.

To address these challenges, there is a pressing need for structured educational frameworks that integrate authentic ophthalmic imaging datasets with end-to-end intelligent analysis workflows in an accessible manner. Interactive and task-oriented learning environments can promote active engagement and facilitate deeper comprehension by enabling learners to directly manipulate data, models, and evaluation metrics. However, dedicated platforms specifically tailored to ophthalmic image analysis education remain limited. Existing tools often lack domain-specific workflow integration, systematic interpretability support, and objective mechanisms for evaluating learning effectiveness.

In this study, we designed and implemented a web-based teaching platform for intelligent ophthalmic image analysis. Unlike general medical imaging AI teaching platforms, the present platform was specifically designed around two representative ophthalmic modalities, fundus photography and OCT, which differ in image characteristics, lesion presentation, and clinical interpretation. The system guides students through a complete learning pipeline encompassing medical image understanding, data preprocessing, model construction, model training, performance evaluation, and result interpretation. In addition to conventional algorithm execution, the platform incorporates an AI-based instructional agent to assist in interpreting analytical outputs. By lowering the operational barrier of algorithm implementation, enhancing process transparency through visualization modules, and providing guided interpretative support, the platform aims to strengthen students’ ability to apply intelligent methods to authentic ophthalmic imaging tasks.

Beyond the platform, we designed a structured experimental teaching scheme that covers the complete intelligent ophthalmic image analysis workflow and integrates pre-class preparation, in-class guided experimentation, and post-class consolidation activities. This structured pedagogical design ensures continuity across learning stages and facilitates progressive knowledge integration throughout the image analysis pipeline. Furthermore, a comprehensive evaluation framework combining objective and subjective measures was employed to assess the educational effectiveness of the proposed platform. The learning outcomes of students in the experimental group were compared with those of a control group using objective performance indicators. In addition, questionnaire-based surveys were conducted to evaluate students’ perceptions of platform usability and self-reported competency improvement. Through this mixed-method evaluation framework, we systematically examined the effectiveness of the proposed platform and the associated teaching design in supporting intelligent medicine education in ophthalmic image analysis.

## 2 Method

### 2.1 System design and development

**Figure 1:**
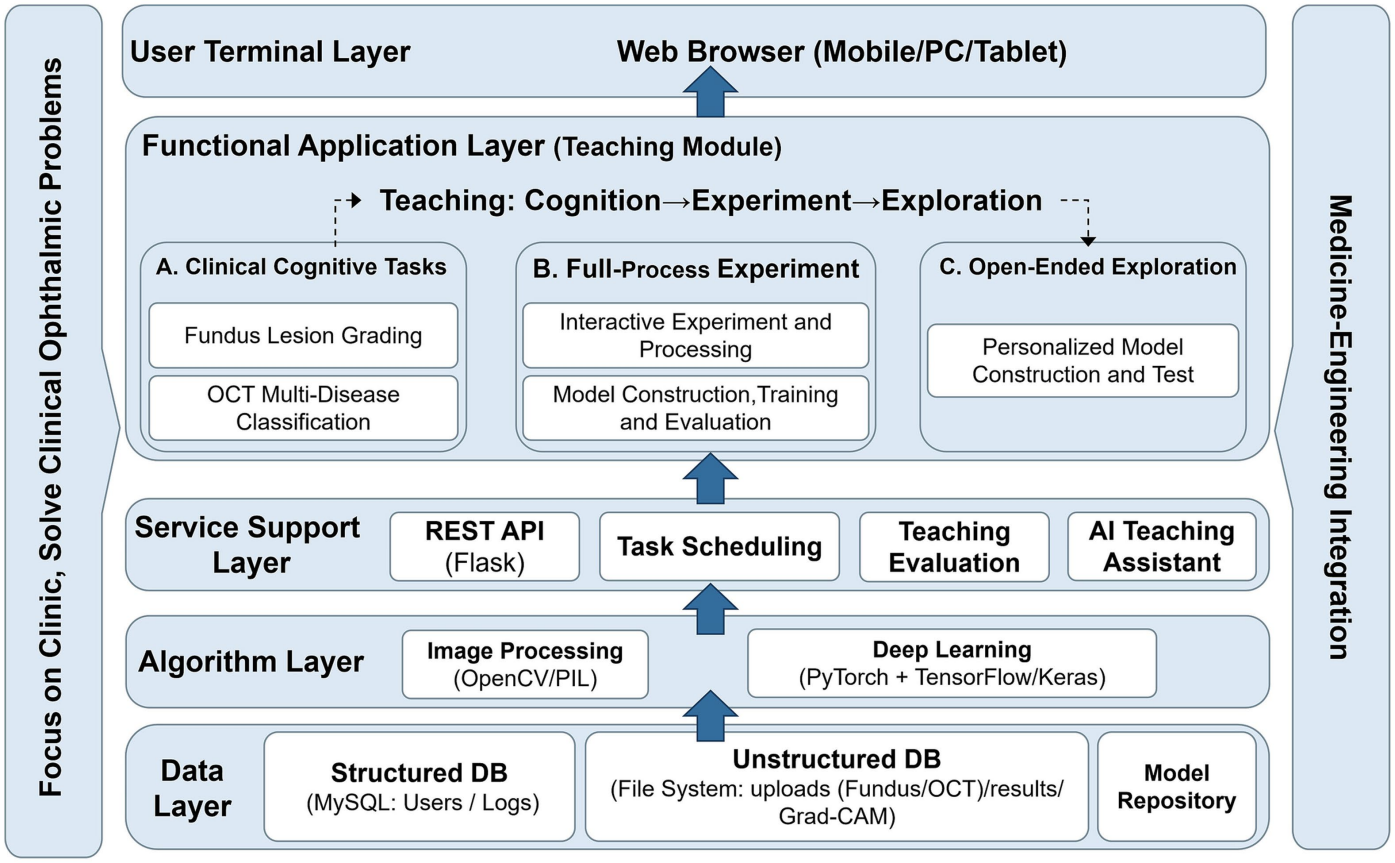
Overall architecture of the system. OCT, optical coherence tomography; REST API, representational state transfer application programming interface; OpenCV, open source computer vision library; PIL, Python Imaging Library; DB, database; Grad-CAM, gradient-weighted class activation mapping.

The proposed platform was developed to support intelligent ophthalmic image analysis education within authentic clinical application contexts. With the objective of integrating ophthalmic medical imaging and artificial intelligence-driven analytical workflows into a unified instructional environment, a front-end/back-end separated architecture and modular software design strategy were adopted. The back-end system was implemented in Python using the Flask framework and integrates mainstream deep learning libraries, including TensorFlow/Keras and PyTorch. These components support ophthalmic image preprocessing, model training and evaluation, inference analysis, and model interpretability functions. The front-end interface was developed using Vue 3 and Vite, incorporating interactive visualization components to enable real-time display of training processes, prediction results, and performance metrics. The overall system architecture is illustrated in Figure 1. The architecture consists of five interconnected layers: the data layer, algorithm layer, service support layer, functional application layer, and user terminal layer. The details of each layer are described below.

The data layer serves as the foundational infrastructure of the system and comprises a structured database, unstructured image database, and a model repository. The structured database manages user information, teaching logs, and experimental records, ensuring traceability and reproducibility of instructional activities. The unstructured database maintains fundus and OCT images, experimental outputs, and interpretability analysis results, thereby providing essential data resources for algorithm training and subsequent instructional analysis. The algorithm layer integrates image preprocessing modules with deep learning-based analytical capabilities. By coordinating traditional image processing techniques with modern deep learning frameworks, this layer supports feature extraction, model training, inference, and performance computation for ophthalmic image tasks. The service support layer exposes unified interfaces through RESTful APIs and incorporates task scheduling, teaching evaluation mechanisms, and an AI-based instructional agent. It enables teaching workflow management, experimental process control, and interpretative assistance for analytical outputs. Functioning as an intermediary layer, it translates algorithmic operations into structured instructional interactions. The functional application layer represents the core pedagogical component of the system. It guides students through clinical cognition tasks, structured end-to-end experiments, and exploratory learning activities. Representative instructional tasks include fundus lesion grading, OCT multi-disease classification, model construction, and performance evaluation. Through progressive task engagement, students develop systematic competence spanning medical image interpretation to intelligent model application. The system further supports personalized model construction and validation, thereby promoting independent experimentation and analytical reasoning. At the user terminal layer, the system is deployed using web-based technologies, enabling access through standard browsers on mobile devices, personal computers, and tablets without additional client installation. This deployment strategy enhances accessibility and instructional flexibility.

**Figure 2:**
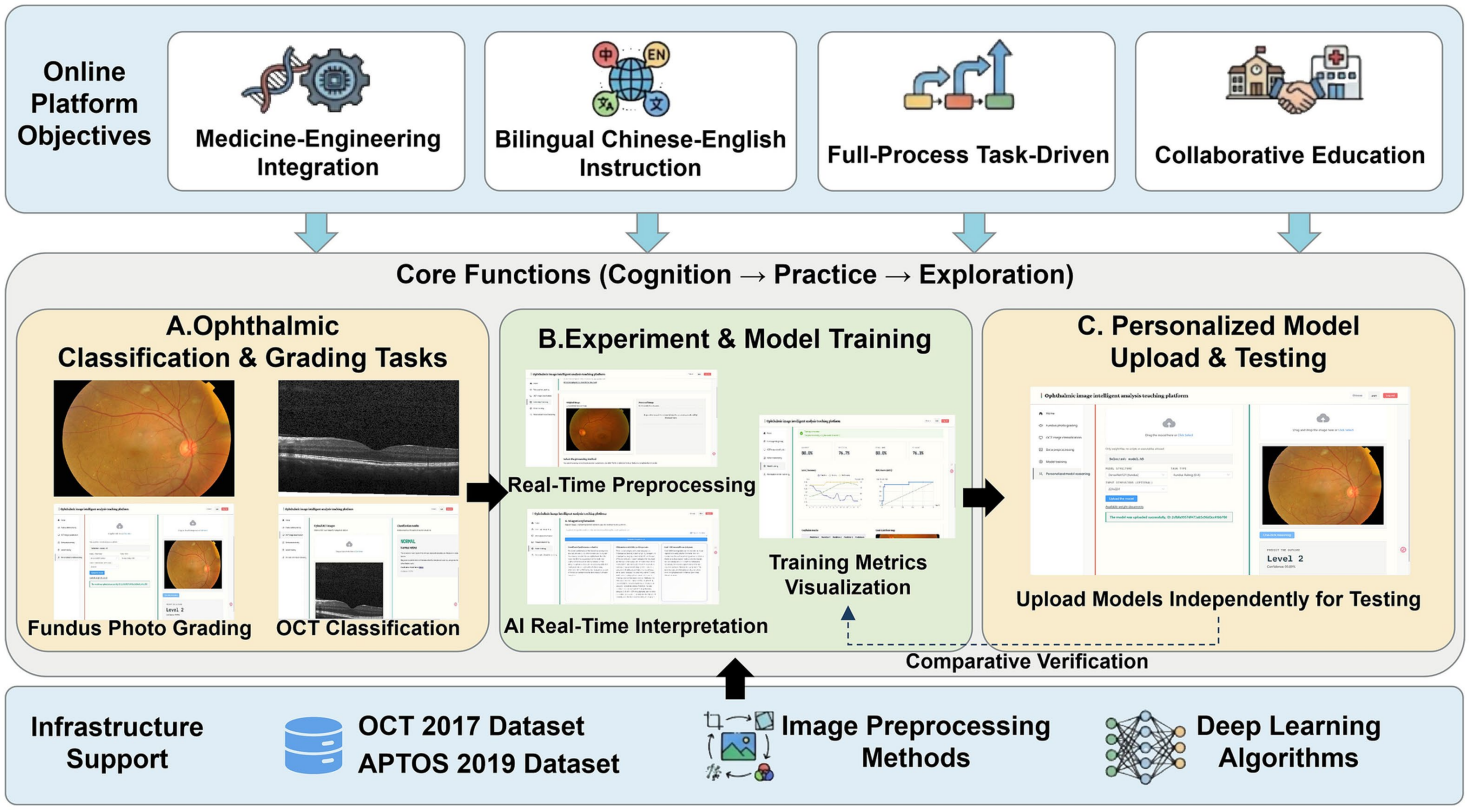
Instructional objectives and functional implementation. OCT, optical coherence tomography; AI, artificial intelligence.

The instructional objectives and functional implementation of the platform are illustrated in Figure 2. The objective of the platform is to emphasize the integration of medical and engineering knowledge. Bilingual support (Chinese and English) is provided to accommodate students from different language backgrounds. Teaching content was organized using a task-driven framework: fundus tasks emphasized lesion visualization, color and texture changes, and anatomical landmarks, whereas OCT tasks emphasized denoising, layered retinal structures, and subtle microstructural abnormalities. In terms of functional implementation, following the “cognition-practice-exploration” pathway, the teaching content is structured into three progressive modules. During the clinical cognition stage, the platform provides representative tasks such as fundus image grading and OCT multi-disease classification, guiding students to understand the relationship between imaging features and disease semantics and to establish foundational clinical image interpretation skills. During the practice stage, the platform supports a complete experimental workflow from data preprocessing to model training and evaluation. Through preprocessing visualization, training monitoring, performance metric display, and AI-assisted explanation, students develop systematic understanding of intelligent ophthalmic image analysis and model behavior. In the exploration stage, the platform enables personalized model construction and testing, allowing students to upload and validate self-developed models and compare different architectures and parameter settings, thereby enhancing independent exploration ability. The platform integrates representative public ophthalmic datasets, preprocessing methods, and deep learning algorithms as the foundational support for instructional activities. Two public datasets used are OCT 2017 Dataset^1^ and APTOS 2019 Dataset.^2^ Through the alignment of instructional objectives and functional modules, the platform establishes a continuous teaching loop from medical image cognition to intelligent model application, supporting a task-driven interdisciplinary teaching model.

### 2.2 Experimental teaching design

**Figure 3:**
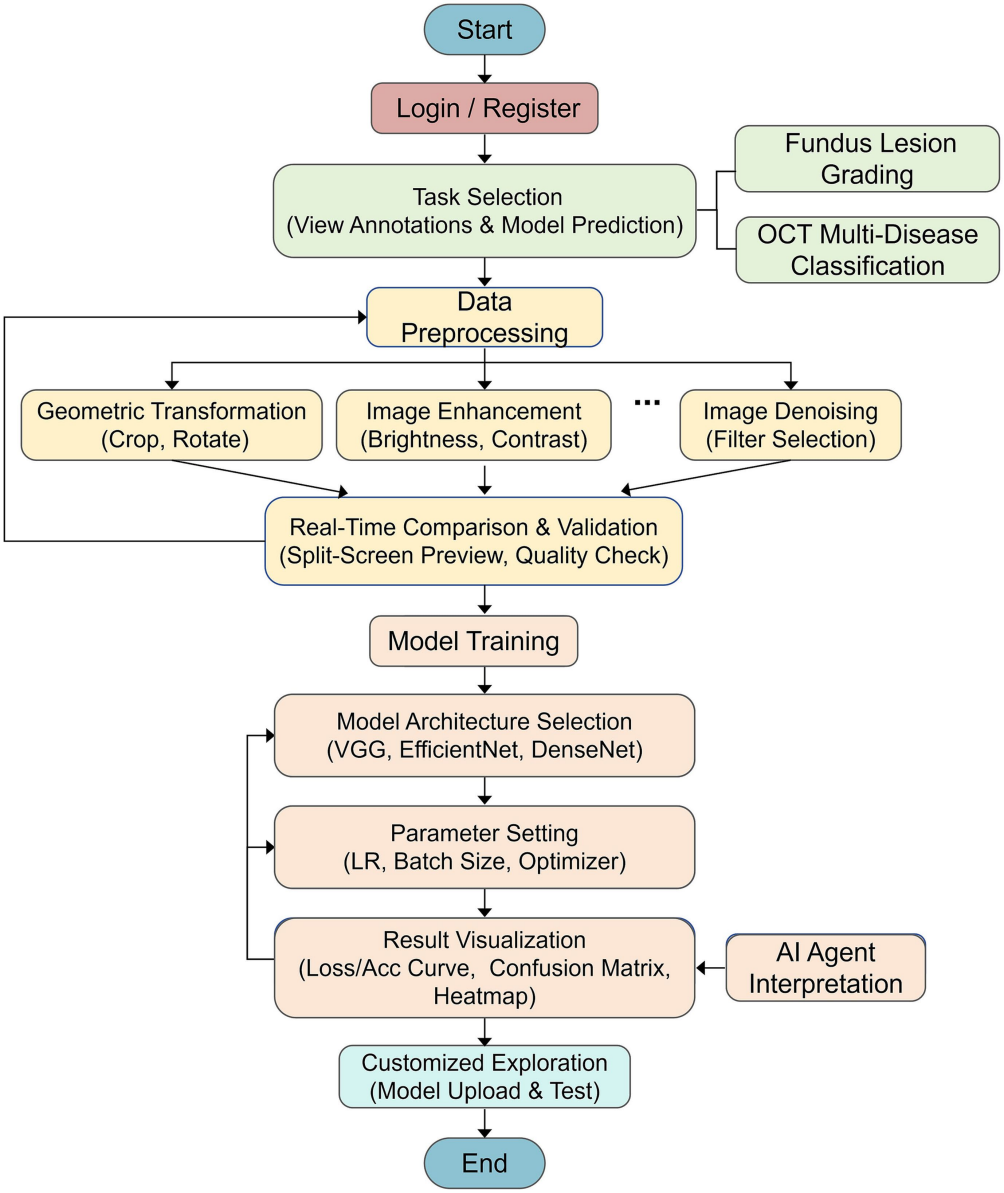
Ophthalmic image analysis of experimental workflow. VGG, visual geometry group; DenseNet, densely connected convolutional neural network; LR, learning rate; Acc, accuracy.

To cultivate students’ ability to apply artificial intelligence methods to medical image analysis in real ophthalmic scenarios, a structured experimental teaching scheme was developed based on the proposed online teaching platform. The scheme covers the complete image processing workflow and integrates pre-class, in-class, and post-class activities. The experimental design considers the learning characteristics of students from different backgrounds, including intelligent medicine, optometry and ophthalmic medicine, interdisciplinary medical-engineering majors. Through a task-driven and iterative approach, students are guided to progressively complete the learning process from medical image cognition to model construction and application. The overall teaching workflow and experimental procedure are illustrated in Figure 3.

The platform-based experimental teaching is organized around the typical workflow of intelligent ophthalmic image analysis. After logging into the system and selecting assigned tasks, students enter specific experimental modules, including representative problems such as fundus lesion grading and OCT multi-disease classification. At the initial stage of the experiment, students observe task-related annotations and model prediction results to establish an overall understanding of ophthalmic image data and task objectives. They then proceed to the data preprocessing stage, where images before and after preprocessing are visually presented to help students understand the impact of preprocessing strategies on analytical outcomes. During the model training phase, students select different network architectures, configure training parameters, and monitor performance changes throughout the training process to complete model construction and optimization. Experimental results are visualized in the form of loss curves, accuracy curves, confusion matrices, and heatmaps to enable model evaluation. An AI-based instructional agent provides explanations of experimental results to enhance students’ understanding and reflection. Finally, the platform supports personalized model construction and independent testing, enabling open-ended exploration and comparative validation.

**Figure 4:**
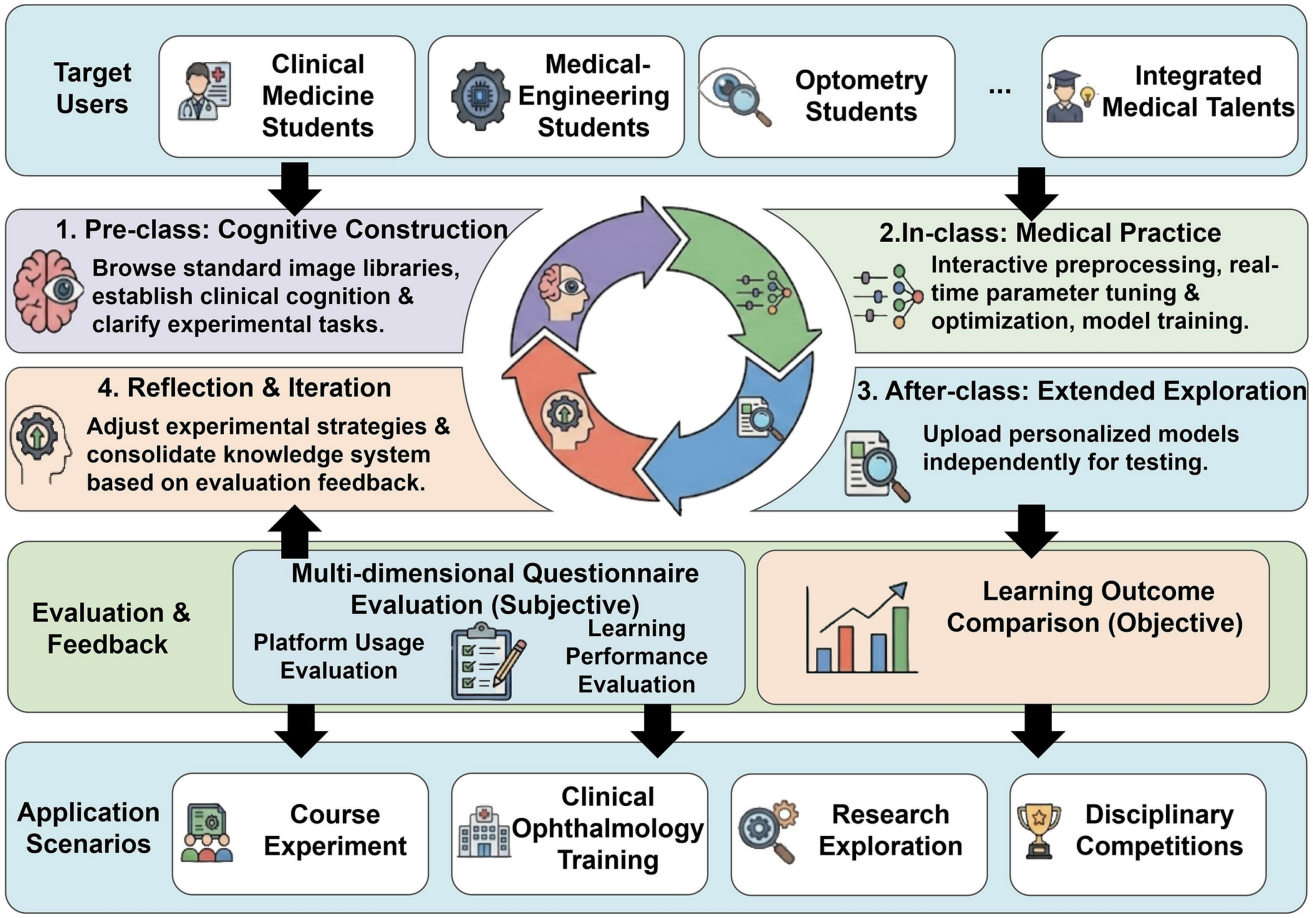
Teaching and application practice.

An integrated experimental teaching model combining pre-class, in-class, and post-class activities was implemented based on the platform, as shown in Figure 4. In the pre-class stage, students review standardized ophthalmic image examples and task descriptions through the platform to develop preliminary understanding of imaging features, disease categories, and experimental objectives, thereby establishing a foundation for subsequent experimental activities. During the in-class stage, interactive experimentation serves as the core activity. Under instructor guidance, students complete key steps including image preprocessing, model training, and parameter adjustment, while observing real-time visualizations of experimental processes and outcomes to reinforce understanding of the intelligent analysis workflow. In the post-class stage, the platform enables personalized model upload and testing, encouraging students to conduct independent exploration based on previous experimental results and to compare different models or strategies. Finally, the educational effectiveness of the platform is evaluated using multi-dimensional subjective questionnaires and objective academic performance indicators. The evaluation results are further used to optimize platform functions and improve instructional design.

### 2.3 Evaluation and questionnaire survey design

In this study, we established a comprehensive evaluation method integrating both subjective and objective assessments. By integrating targeted questionnaire surveys and a controlled teaching experiment, the instructional value of the platform was examined from two dimensions: user experience and learning outcomes.

The subjective evaluation was conducted primarily through questionnaire surveys. Participants included students from multiple majors, such as optometry and ophthalmic medicine, intelligent medicine engineering, biomedical engineering, and clinical medicine. Questionnaires were distributed and collected online communication channels (e.g., WeChat and QQ groups) in an anonymous format and included system usability evaluation and competency ratings. System usability was assessed using the classical System Usability Scale (SUS) with a five-point Likert scale (21). And competency ratings covered five dimensions: medical image analysis, AI ethics awareness, AI modeling capability, model result interpretation, and interdisciplinary confidence. Before formal administration, reliability and construct validity analyses were performed, showing high internal consistency (Cronbach’s *α* = 0.923) and acceptable construct validity, as supported by a KMO value of 0.908 and a significant Bartlett’s test of sphericity (*χ*^2^ = 632.75, *p* < 0.001).

The inclusion criteria were as follows: (1) completion of all operational modules on the platform; and (2) complete questionnaire submission. The exclusion criteria were as follows: (1) missing responses in key items; (2) excessively short response time (<2 min); and (3) invalid questionnaires showing patterned responding. A total of 121 valid questionnaires were finally included in the analysis. Because participation in the questionnaire survey was voluntary, the possibility of self-selection bias could not be completely excluded.

For objective evaluation, 64 third-year undergraduate students majoring in Intelligent Medicine Engineering at Southwest Medical University were recruited as the study population. All participants were students from the same teaching class, and were assigned to the control group or the experimental group using sex-stratified randomization, with 32 students in each group. To reduce the influence of potential confounding factors, both groups were taught by the same instructor and followed the same teaching syllabus, teaching duration, course content, and assessment requirements. To ensure objective assessment, the blind method was implemented for assessment raters. The platform-assisted intervention in the experimental group was conducted over a period of more than 8 weeks. The control group received traditional lecture-based teaching only, whereas the experimental group received the same traditional teaching supplemented by platform-assisted learning activities.

The comprehensive course assessment focused on students’ mastery of core knowledge, including medical image cognition and application of intelligent analysis methods, as well as their ability to transfer theoretical knowledge to practical medical image analysis problems. Learning outcomes between the experimental and control groups were compared to evaluate instructional effectiveness.

### 2.4 Statistical analysis

All statistical analyses were performed using Python 3.9. The Shapiro–Wilk test was used to assess the normality of continuous variables. The results show that regarding the comprehensive course assessment, comprehensive competence and theoretical competence were normally distributed, whereas practical competence was non-normally distributed. And all other continuous variables, including SUS scores and competency ratings, were also skewed. Normally distributed data were presented as the mean ± standard deviation, and independent samples t-test was used for between-group comparisons. Non-normally distributed data were expressed as median [M (P25, P75)] and analyzed using the Mann–Whitney *U* test. *Post-hoc* statistical power analysis was conducted based on the observed effect size (Cohen’s *d* = 0.81) and sample size (*n* = 32 per group) in the controlled experiment. At a two-sided *α* level of 0.05, the achieved statistical power was 88.97%, which exceeded the conventional standard of 80%, indicating that the sample size of this study was sufficient to effectively detect between-group differences. The total SUS score was calculated according to the standard scoring procedure, ranging from 0 to 100, with 68 regarded as the benchmark for acceptable usability (22). A one-sample Wilcoxon signed-rank test was used to compare the observed SUS scores with the benchmark value. Differences in SUS scores among students from different majors were assessed using the Kruskal–Wallis *H* test, followed by Dunn’s *post-hoc* multiple pairwise comparisons with Bonferroni correction to identify the sources of between-group differences. A *p-*value <0.05 was considered statistically significant.

## 3 Results

### 3.1 System implementation

**Figure 5:**
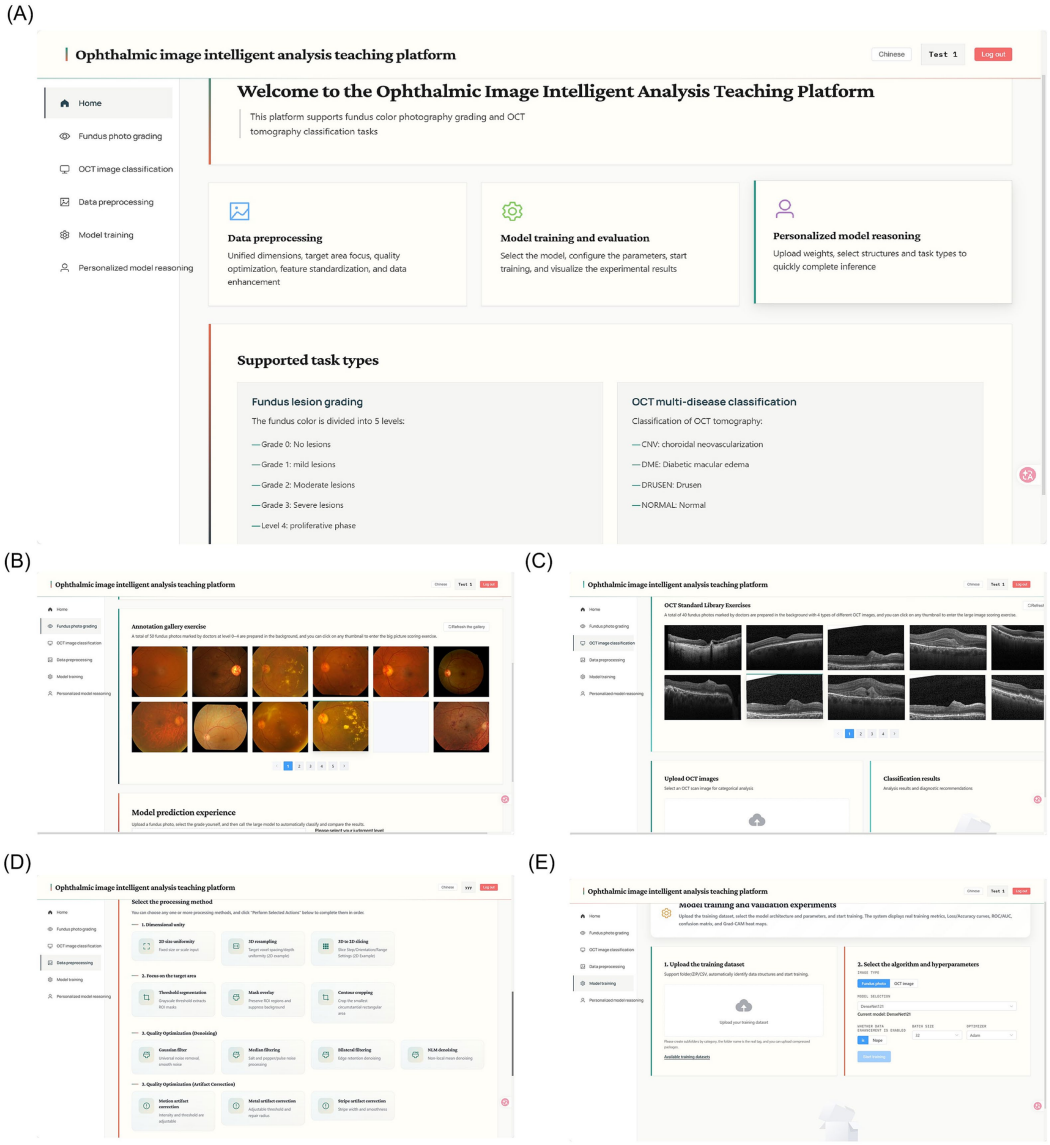
Intelligent ophthalmic image analysis teaching platform. **(A)** Homepage of the teaching platform. **(B)** Fundus lesion grading interface. **(C)** OCT multi-disease classification interface. **(D)** Data preprocessing interface. **(E)** Model training interface.

**Figure 6:**
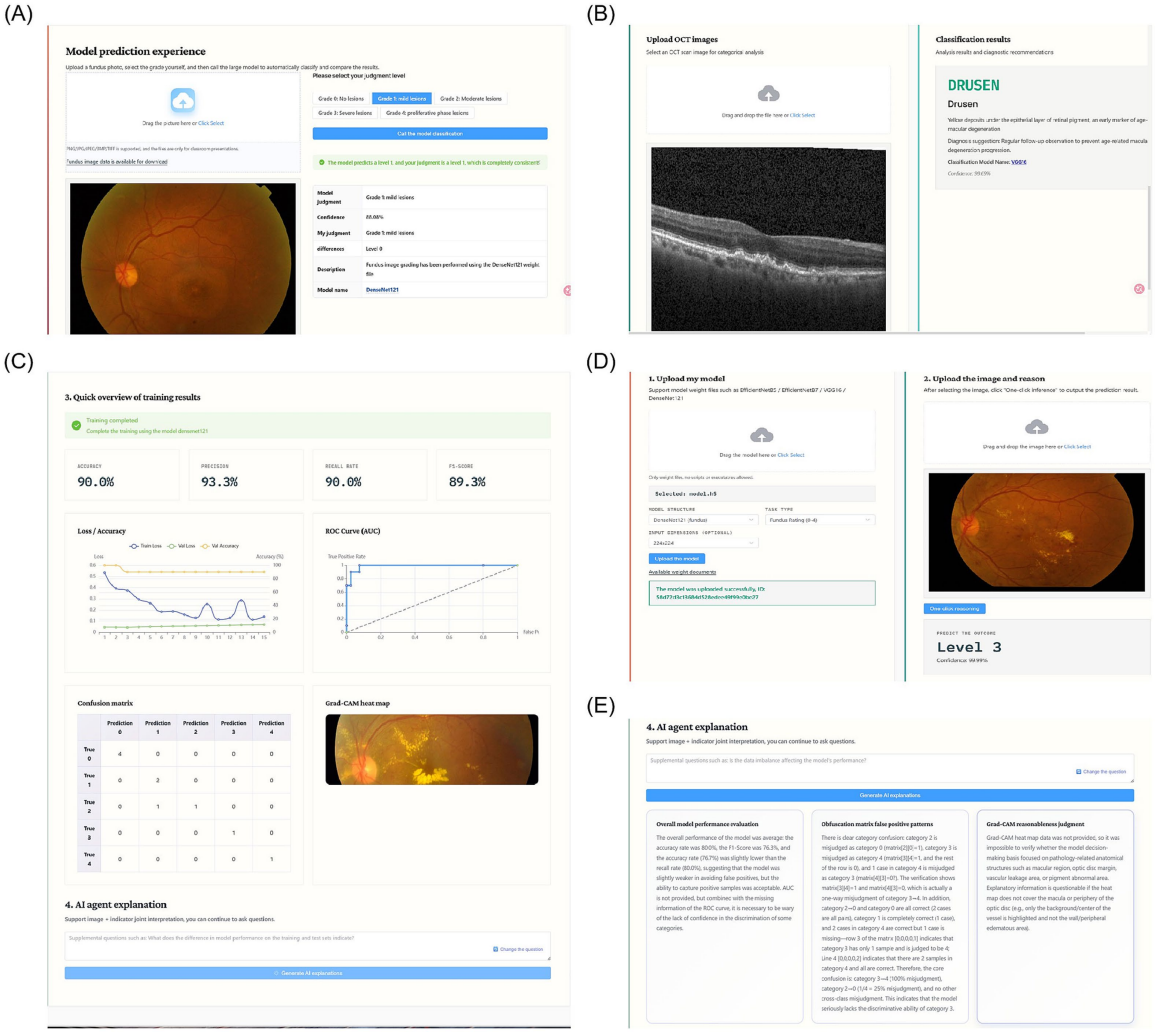
Representative experimental results. **(A)** Fundus lesion grading results. **(B)** OCT multi-disease classification results. **(C)** Model performance after training. **(D)** Personalized model testing results. **(E)** AI-assisted interpretation of analytical results.

The platform was developed using a web-based front-end/back-end separation architecture to support intelligent ophthalmic medical image analysis instruction. The system focuses on two representative ophthalmic imaging modalities: fundus photography and optical coherence tomography (OCT), and establishes a complete instructional workflow covering the full process of intelligent medical image analysis. The main interfaces and functional modules of the platform are shown in Figure 5. The system supports multi-format medical image upload, visualized parameter configuration, and interpretative assistance for analytical results, forming a structured learning pathway from ophthalmic clinical image cognition to intelligent model application. Representative experimental results of each core module are visualized in Figure 6.

In addition to functional implementation, the platform was deployed on a routine teaching server, and basic technical performance testing was conducted. The results showed that the registration interface maintained success rates of 99.13–99.71% under concurrent loads of 10, 30, and 50 users, and that the 95th percentile (P95) response time of major system interfaces ranged from 3.24 to 4.69 s. In single-image processing tests, the average processing time was 0.66 s for OCT images and 2.14 s for fundus images, with corresponding P95 values of 1.81 s and 6.03 s, respectively. These results suggest that the platform can support concurrent multi-user access and routine teaching use under normal instructional conditions.

### 3.2 Student evaluation of the ophthalmic teaching platform

**Figure 7:**
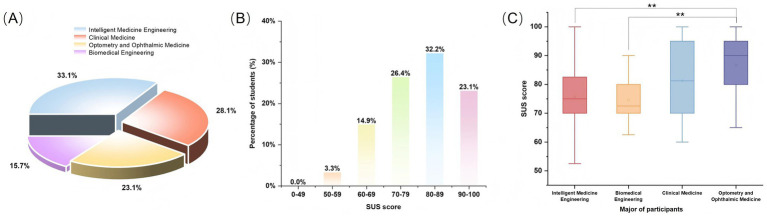
Distribution of survey participants and results of the SUS assessment. **(A)** Proportion of participants by major: students participating in the survey were from four majors, namely Intelligent Medicine Engineering (33.1%), Clinical Medicine (28.1%), Optometry and Ophthalmic Medicine (23.1%), and Biomedical Engineering (15.7%). **(B)** Distribution of SUS score grades: most students’ SUS scores were concentrated in the 80–89 range (32.2%), followed by 70–79 (26.4%) and 90–100 (23.1%). Only 3.3% of students scored in the 50–59 range, and no student scored below 50. **(C)** Comparison of SUS scores across majors: students majoring in Optometry and Ophthalmic Medicine achieved the highest median SUS score, which was significantly higher than that of students in Intelligent Medicine Engineering and Biomedical Engineering (*p* < 0.01).

The results of the questionnaire survey are presented in Figure 7. A total of 121 valid questionnaires were collected. As shown in Figure 7A, 40 students (33.1%) majored in Intelligent Medicine Engineering, 34 (28.1%) in Clinical Medicine, 28 (23.1%) in Optometry and Ophthalmic Medicine, and 19 (15.7%) in Biomedical Engineering.

Figure 7B shows that more than half of the students (55.3%) obtained scores above 80, whereas only 3.3% scored less than 60. As shown in Table 1, the mean SUS score across all respondents was 80.10 ± 12.57, the median was 80.0 (70.0, 87.5), with 81.8% of students achieving scores above 68. The median SUS score was significantly higher than the benchmark value of 68 (*p* < 0.001). Students majoring in Optometry and Ophthalmic Medicine achieved the highest median SUS score [90.0 (80.0, 95.0)], followed by Clinical Medicine [81.2 (70.0, 95.0)], Intelligent Medicine Engineering [75.0 (71.2, 82.5)], and Biomedical Engineering [72.5 (70.0, 80.0)]. The proportion of students obtaining SUS scores above 68 was 89.3% in Optometry and Ophthalmic Medicine, 85.3% in Clinical Medicine, 78.9% in Biomedical Engineering, and 75.0% in Intelligent Medicine Engineering. Significant differences were observed among students from different majors (*p* < 0.001). Figure 7C illustrates the SUS scores of Optometry and Ophthalmic Medicine students were significantly higher than those of Intelligent Medicine Engineering (*p* = 0.0035) and Biomedical Engineering (*p* = 0.0031) students.

**Table 1:** Comparison of SUS scores among different majors.

Majors	Number of participants	SUS score [Median (P25, P75)]	SUS score (mean ± SD)	SUS score more than 68 (%)	Kruskal–Wallis *H* test
Optometry and Ophthalmic Medicine	28	90.0 (80.0, 95.0)	86.70 ± 10.76	25 (89.3%)	*H* = 16.522 (*p* < 0.001)
Clinical Medicine	34	81.2 (70.0, 95.0)	81.25 ± 12.74	29 (85.3%)	
Intelligent Medicine Engineering	40	75.0 (71.2, 82.5)	75.69 ± 11.08	30 (75.0%)	
Biomedical Engineering	19	72.5 (70.0, 80.0)	74.61 ± 7.60	15 (78.9%)	
Total	121	80.0 (70.0, 87.5)	80.10 ± 12.57	99 (81.8%)	

**Figure 8:**
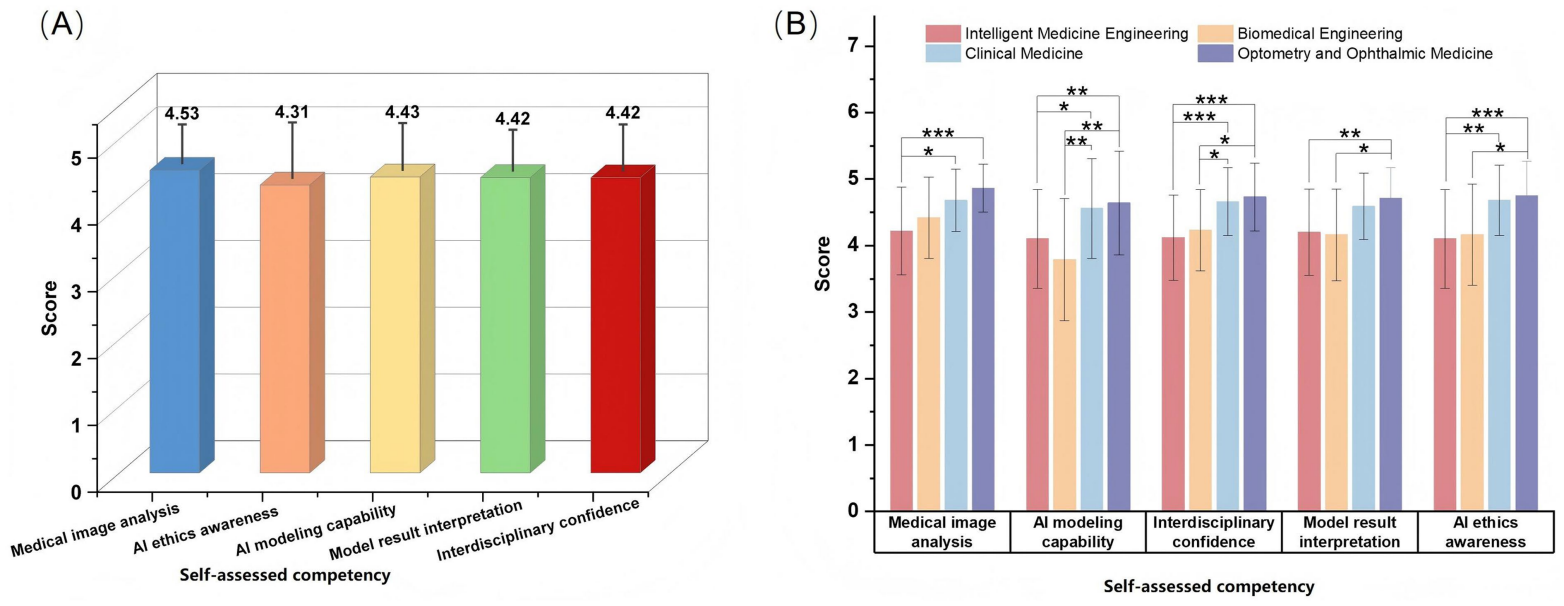
Results of self-assessed competency improvement. **(A)** Self-assessed competency improvement across five dimensions: all students achieved high self-assessed scores in the five dimensions, with mean scores ranging from 4.31 to 4.53. **(B)** Comparison of self-assessed competency scores across different majors: students from medical background majors (Clinical Medicine, Optometry, and Ophthalmic Medicine) achieved significantly higher scores than engineering-oriented students (Intelligent Medicine Engineering, Biomedical Engineering) in all five dimensions, with students majoring in Optometry and Ophthalmic Medicine scoring the highest in each dimension (^*^p < 0.05, ^**^p < 0.01, and ^**^p < 0.001).

The competency ratings across the five dimensions are shown in Figure 8A. Overall, students reported relatively positive improvement in all dimensions, with most mean scores exceeding 4.0 on the 5-point scale. However, as shown in Figure 8B, significant differences across majors were observed in multiple dimensions (*p* < 0.05). Students in Optometry and Ophthalmic Medicine and Clinical Medicine generally showed higher scores, whereas students in Intelligent Medicine Engineering and Biomedical Engineering tended to report relatively lower scores. These findings indicate that, although the platform was positively perceived overall, the extent of perceived competency improvement varied according to disciplinary background.

### 3.3 Comparison of learning outcomes between the experimental and control groups

**Figure 9:**
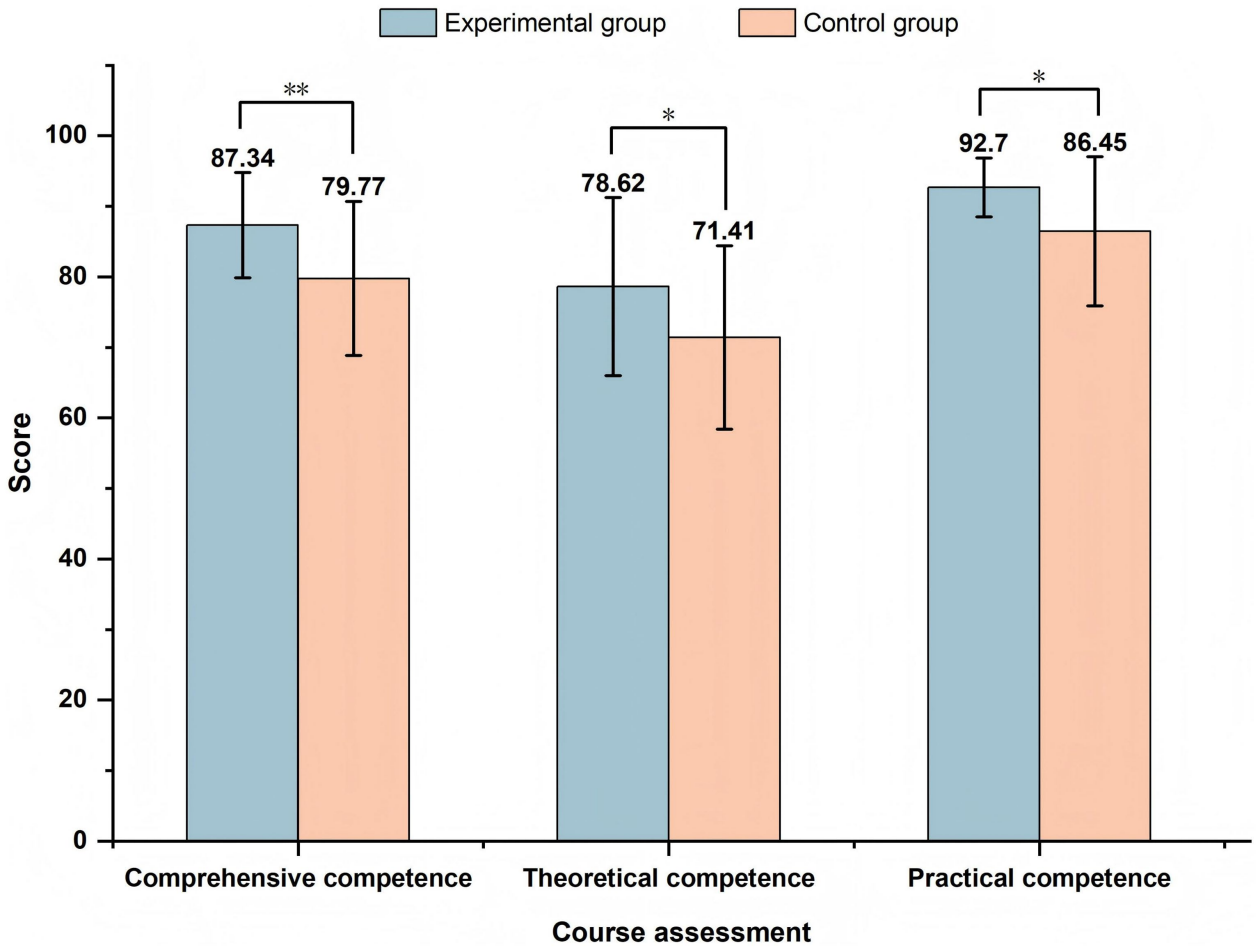
Comparison between the experimental and control groups. The experimental group scored significantly higher than the control group in all three dimensions (^*^p < 0.05 and ^**^p < 0.01).

As shown in Figure 9, the experimental group achieved higher scores than the control group across all evaluated dimensions. For comprehensive competence, the experimental group obtained a mean score of 87.34 ± 7.45, compared with 79.77 ± 10.94 in the control group, and the difference was statistically significant (*p* < 0.01). In terms of theoretical competence, the experimental group achieved 78.62 ± 12.62, whereas the control group scored 71.41 ± 13.03. The difference was statistically significant (*p* < 0.05). The experimental group demonstrated superior practical competence, with a mean score of 92.70 ± 4.18 compared to 86.45 ± 10.57 in the control group, and the difference was statistically significant (*p* < 0.05).

## 4 Discussion

With the expanding role of artificial intelligence in ophthalmic training and clinical decision support, there is an increasing need for structured educational models that integrate AI-driven image analysis into medical curricula (23, 24). Previous studies have suggested that a major challenge in medical AI education is the gap between clinical understanding and algorithmic learning (23, 25, 26). This issue may be more evident in ophthalmic education, where the interpretation of fundus and OCT images depends not only on image-based analysis, but also on the understanding of lesion characteristics and clinical semantics (27). In this context, the present study developed and evaluated a web-based intelligent ophthalmic image analysis teaching platform and examined its usability, perceived competency enhancement, and instructional effectiveness through a controlled teaching experiment. The results suggest that the platform is a feasible teaching tool for intelligent ophthalmic image analysis and may provide a preliminary reference for ophthalmic AI education and training.

The overall SUS score of our platform was significantly higher than 68, indicating a favorable level of usability (28). More than 80% of respondents obtained scores above the benchmark, suggesting broad acceptance among students from diverse academic backgrounds. The SUS score of our platform (80.10 ± 12.57) was slightly higher than that reported for FunduScope (76 ± 6.79) (29), an ML-based interactive tool developed for training junior ophthalmologists in diabetic retinopathy detection, and lower than that reported for the Retina e-Learning App (87.7 ± 11.9) (30), an ophthalmic e-learning application evaluated among ophthalmologists. These comparisons should be interpreted cautiously because the learner populations, teaching objectives, and task designs differed across studies. Nevertheless, these studies collectively suggest that ophthalmic digital learning tools can achieve favorable usability, and that platform acceptance is an important prerequisite for supporting image-based ophthalmic training.

The differences in both SUS scores and competency ratings across majors suggest that disciplinary background influenced students’ perceptions of platform usability and their perceived improvement across the five competency dimensions. The higher scores among students in medical background may reflect their greater familiarity with fundus and OCT images, lesion characteristics, and related clinical semantics, which likely reduced the cognitive burden during platform use. By contrast, students from engineering-oriented backgrounds, such as Intelligent Medicine Engineering and Biomedical Engineering, despite having stronger technical foundations, may have found it more difficult to connect ophthalmic image interpretation with platform-based AI learning because of their more limited clinical background. In addition, the current platform emphasizes structured task workflows and instructional guidance, whereas engineering-oriented students may expect greater technical flexibility and deeper algorithmic exploration. This is consistent with previous studies suggesting that medical AI education needs to balance clinical understanding with technical learning, and that learners from different academic backgrounds may show different educational needs (23, 31). These findings suggest that future platform optimization should provide more differentiated support, particularly by strengthening guidance on the clinical semantics of ophthalmic images and developing layered learning pathways for students from different academic backgrounds.

Consistent with previous studies, usability and perceived usefulness of educational platforms are closely associated with learner engagement and academic performance (32–34). In the present study, all five competency dimensions showed mean scores above 4.0, suggesting that the platform may support students’ understanding of ophthalmic image analysis and AI-related learning tasks. This is in line with the growing emphasis on integrating AI competencies into medical education (31, 35, 36). In particular, the relatively high score in AI modeling capability suggests that the platform may help students better understand how AI methods are applied to medical image analysis (37–39). In additional, the higher scores in theoretical, practical, and comprehensive competence in the experimental group suggest that platform-assisted learning may support both knowledge acquisition and practical application in ophthalmic image analysis teaching. This advantage may be related to the structured learning process provided by the platform, which integrates key steps from image preprocessing to model evaluation within a single teaching workflow.

From an instructional design perspective, the staged learning pathway (cognition-practice-exploration) may help students progressively integrate clinical understanding with computational modeling. The incorporation of AI-assisted interpretative feedback potentially reduces abstraction in deep learning processes by contextualizing model outputs within medical semantics. Several limitations should be acknowledged. First, although the questionnaire survey included students from multiple disciplines, the controlled experiment was conducted only among students majoring in Intelligent Medicine Engineering, which may limit the generalizability of the findings. Subgroup analysis was not performed to further examine differences between learners with medical and engineering backgrounds. Second, detailed feedback on individual functional modules and operational pain points was not systematically collected, which limited the identification of specific directions for platform optimization. Third, the current platform mainly serves teaching and basic training, and its integration with authentic clinical ophthalmic practice remains limited. Teacher-side user experience was also not systematically evaluated. Finally, although several teaching conditions were kept consistent between groups, instructional factors could not be fully standardized, and the influence of differences in practice exposure cannot be completely excluded.

## 5 Conclusion

This study developed and evaluated a web-based intelligent ophthalmic image analysis teaching platform that integrates authentic clinical imaging data with structured AI-driven analytical workflows. The platform supports end-to-end instructional processes, including image cognition, preprocessing, model training, evaluation, and interpretative assistance. The evaluation results demonstrated high system usability, positive self-reported competency improvement, and significantly enhanced learning outcomes. These findings suggest that integrating clinically contextualized AI experimentation into ophthalmic education may facilitate interdisciplinary knowledge integration and improve applied analytical competence. Overall, the proposed platform provides a feasible instructional framework for intelligent ophthalmic image analysis and may serve as a preliminary reference for ophthalmic AI education and training.

## Data Availability

The raw data supporting the conclusions of this article will be made available by the authors, without undue reservation.

## Footnotes

1
https://www.kaggle.com/paultimothymooney/kermany2018

2
https://www.kaggle.com/c/aptos2019-blindness-detection
